# The mechanisms of mutual relationship between malignant hematologic cells and mesenchymal stem cells: Does it contradict the nursing role of mesenchymal stem cells?

**DOI:** 10.1186/s12964-022-00822-6

**Published:** 2022-03-02

**Authors:** Alireza Goodarzi, Mohsen Valikhani, Fatemeh Amiri, Armita Safari

**Affiliations:** 1 Department of Medical Laboratory Sciences, School of Paramedicine, Hamadan University of Medical Sciences, Shahid Fahmideh Blvd., The Opposite Side of Mardom Park, Hamadan, 6517838741 Iran; 2 Hematology Department, School of Allied Medical Science, Iran University of Medical Sciences, Tehran, Iran; 3 Student Research Committee, Hamadan University of Medical Science, Hamadan, Iran

**Keywords:** Mesenchymal stem cell, Hematologic malignancy, Tumor

## Abstract

Mesenchymal stem/stromal cells (MSCs) are known as the issue in biology because of some unpredictable characteristics in the different microenvironments especially in their bone marrow niche. MSCs are used in the regenerative medicine because of their unique potentials for trans-differentiation, immunomodulation, and paracrine capacity. But, their pathogenic and pro-survival effects in tumors/cancers including hematologic malignancies are indisputable. MSCs and/or their derivatives might be involved in tumor growth, metastasis and drug resistance in the leukemias. One of important relationship is MSCs and hematologic malignancy-derived cells which affects markedly the outcome of disease. The communication between these two cells may be contact-dependent and/or contact-independent. In this review, we studied the crosstalk between MSCs and malignant hematologic cells which results the final feedback either the progression or suppression of blood cell malignancy.

**Graphical abstract:**

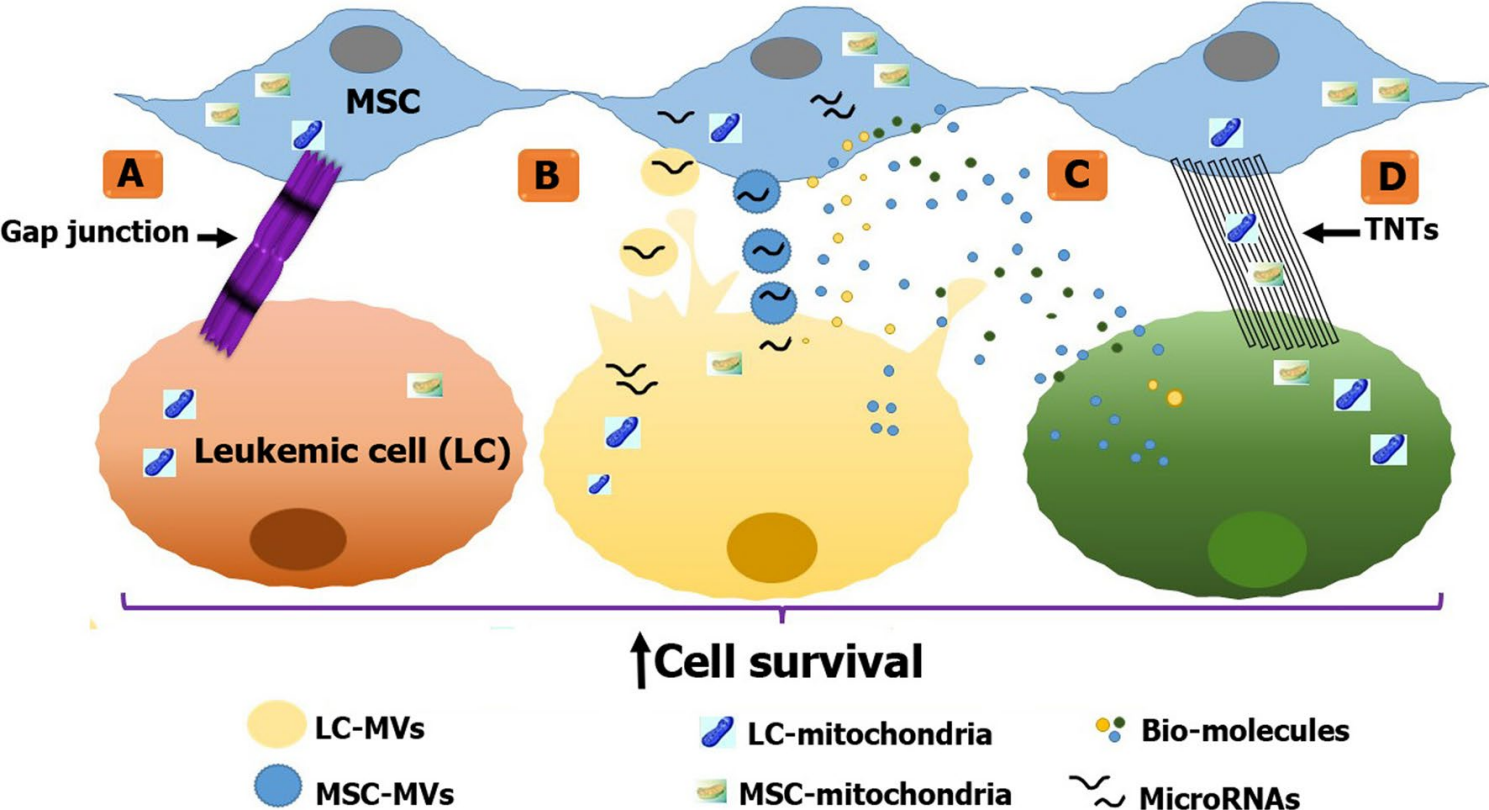

**Video abstract**

**Supplementary Information:**

The online version contains supplementary material available at 10.1186/s12964-022-00822-6.

## Background

Bone marrow-derived mesenchymal stromal cells (BM-MSCs) are a rare population of non-hematopoietic multipotent cells that are recognizable and sortable by being positive for MSC markers such as CD90, CD73 and CD105 and being negative for hematopoietic markers like CD34 and CD45 [1, 2]. It has been revealed that it is not just the physical support provided by MSCs in microenvironment for target cells, but a bidirectional conversation between them is routed [3, 4]. A substantial number of studies declare that BM-MSCs adopt several methods to establish this communication. These mechanisms can be divided into two main categories: contact-dependent including tunneling nanotubes (TNTs) [5, 6], gap junctions [7], and contact-independent including paracrine activity and microvesicle/exosomes delivery through which microRNA (miRNA), immuno-modulatory molecules and mitochondria can be transferred [8–12]. MSCs are mainly being used in the regenerative medicine [13–15]. But in terms of malignancy, it is a subject of controversial that whether MSCs are pro- or anti-tumorigenic [16]. However, to digress from malignancies, MSCs are also used in myocardial infarction (MI) [17]. They play an indirect role in MI by recruitment of macrophages in angiogenesis to promote tissue regeneration [18]. The study of these effects and conversations provides a fertile ground of investigation [19].

Returning to malignancies, although MSCs have shown therapeutic properties mainly by their potentials for trans-differentiation, immunomodulation, and apoptosis induction [2, 20, 21], their pathogenic, leukemogenic and pro-survival effects in hematologic malignancies are indisputable [22, 23]. Cellular mechanisms include RNA processing, ubiquitin–proteasome pathway, cell cycle regulation, cellular stress and non-canonical Wnt signaling are modulated in the leukemia cell lines co-cultured with MSCs [24].

Indeed, MSCs have been suspected of being the culprit of elevated tumor growth, metastasis and drug resistance in the leukemias [25]. Particularly in chronic lymphocytic leukemia (CLL) cells, survival and drug resistance signals as well as CLL-cell trafficking and tissue homing signals are under bone marrow stromal cells (BMSCs) control [26]. Even in a mouse model of the pre-leukemic disorder Schwachman-Diamond syndrome (SDS), genotoxic stress and subsequently DNA damage response (DDR) activation in hematopoietic stem and progenitor cells have been reported as the main consequence of abnormal activity of mesenchymal cells [27]. Therefore, this study covers a lot of ground in this matter and offers a full explanation of mechanisms by which MSCs counter treatment strategies. Herein, the contact-dependent and contact-independent mechanisms that involve in the MSC and leukemic cells conversation are presented.

## Contact-dependent

### Tunneling nanotubes (TNTs)

TNTs are intercellular transient structures of about 50–200 nm in diameter which are made of polymerization of F-actin; thereby, they are naturally vanishing and henceforth uncontrollable. Likewise, biomolecules and cellular organelles can be transported through them [28].

First of all, chemoresistance can be modulated in other solid tumor cells like SKOV3 ovarian cancer cells and MCF7 breast cancer cells as a consequence of preferential transfer of mitochondria from endothelial to cancer cells through TNTs modulates [29]. Pinto and et al. have reviewed that TNT-like connections are used by cancers to modify their potential chemoresistance, migration, metabolism, metastasis and angiogenesis [30].

However, in the leukemias TNTs serve the reliable infrastructure for trafficking both vesicle and protein to leukemic cells [31]. This type of intercellular relationship is forged, and as it has been proven in B cell acute lymphoblastic leukemia (B-ALL), it is not a monologue delivered by MSCs but dialogues can be held. As an example, using TNTs, BM-MSC secretome profile is pervasively converted to a leukemia pro-survival profile in B-lymphoblastic leukemia [32]. Bidirectional cytoplasmic transport has also been observed between MSCs and activated benign T cells which exerts suppressive effect on the proliferation rate and IFN-γ production of T cells [33].

Furthermore, through a TNT routed communication, primary B-cell precursor ALL (BCP-ALL) cells deliver autophagosomes, mitochondria, intercellular adhesion molecule 1 (ICAM1) and other lipophiles to MSCs leading to cytokine secretion, leukemic cell survival and drug resistance [34]. These pro-survival cytokines include interferon-γ-inducible protein 10 (IP-10), CXCL10, IL-8, monocyte chemotactic protein-1 (MCP-1) and CCL2 [35]. Disruption of TNTs would physically affect this support and significantly re-sensitizes BCP-ALL cells to chemotherapeutics like prednisolone [35]. However, being exposed to chemotherapeutic drugs, mitochondrial transfer from Jurkat cells, a T cell acute lymphoblastic leukemia (T-ALL) cell line, to MSCs is promoted. Although, it is primarily conducted by contact-dependent manners including TNTs and gap junctions (GJs), nevertheless microvesicles (contact-independent) are also to some extent responsible for this trafficking [36]. In fact, Jurkat cells exhibit a trafficking arabinoside (ARA-C) or methotrexate. This leads to a substantial promotion of Jurkat cells' survival, due to a dramatic reduction of reactive oxygen species (ROS) levels inside them [36, 37]. The resulting increased production of mitochondrial adenosine triphosphate (ATP), mitochondrial transfer leads to increased chemoresistance [38].

By the same token, in acute myelogenous leukemia (AML), mitochondrial transfer primarily through TNT has been contingently associated with chemoresistance [39]. Both leukemic blasts and leukemia initiating cells of AML are given the privilege of further survival in battle with some chemotherapies by the TNT-routed mitochondrial transfer from MSC [40]. By up to 14%, mitochondrial mass in AML cells increases in co-cultures with MSCs. Consequently, cytotoxic effects of the nucleoside analog ARA-C would be hedged against AML cell lines [40].

To enhance transferring from MSC to AML blasts through TNT, mitochondrial biogenesis in MSC is stimulated by AML-derived NADPH oxidase 2 (NOX2) superoxide. Correspondingly, this facilitation can be taken place in multiple myeloma (MM) cells by CD38 expressing MM cells [41]. Inhibition of NOX2 is characterized by a downward trend in mitochondrial transfer and an upward trend in AML cells apoptosis [42]. Autophagy, on the other hand is increased in AML cells chiefly because autophagosomes transfer from MSCs to AML cells using TNTs [32].

Tyrosine kinase inhibitors and interferon‐α used in chronic myeloid leukemia (CML) cell lines increase TNT formation and cell adhesion [43]. The so-called stroma-mediated imatinib resistance would be resulted from this TNT formation. To clarify, CML cells receive cellular vesicles from and send them out to MSCs through TNTs. These vesicles finally attenuate imatinib mediated caspase activity and thereby apoptosis [44]. Notably in CML, TNTs facilitate exosomes transportation and exosomes in turn stimulate TNT formation; so, they can synergistically interact and multiply their communication [45].

Turning to lymphoma, we must note that malignant B cells upregulate BCL-2 family proteins following receiving soluble factors from MSCs. Activation of the oncogenic pathways like NOTCH1 signaling is also stimulated by MSCs reported in different B cell malignancies, MM and CLL [46, 47]. Examples of MSCs and malignant hematologic cells communications via TNTs have been summarized in Table 1

**Table 1:** Communication between MSCs and hematologic malignancies/cell lines through tunneling nanotubes (TNTs) and its effects

Type of malignancy/cell	Kind of material transferred	Target	Results	References
ALL	Pro-survival cytokines	ALL cells	Conversion to a leukemia pro-survival	[35]
ALL	IL-6/TNFα/IL-1β	ETV6‐RUNX1 harboring cells	DNA damage accumulation	[48]
BCP-ALL	Autophagosomes, mitochondria, ICAM1 and other lipophiles	MSCs	Cytokine secretion, leukemic cell survival, drug resistance	[34]
Jurkat cells	Mitochondria	Mostly MSCs	Leukemic cell survival, ↓ROS, chemoresistance	[36, 37] [38]
AML	Mitochondria	AML cells	Chemoresistance, cytotoxic effects of the nucleoside analog ARA-C	[39]
AML	Autophagosome	AML cells	Autophagy	[32]
AML	NADPH oxidase 2 (NOX2) superoxide	MSCs	Enhances transferring from MSC to AML	[41]
CML	Cellular vesicles	Bi-directional	Imatinib resistance, ↓imatinib mediated caspase activity, apoptosis	[44]
Multiple myeloma	NADPH oxidase 2 (NOX2) superoxide	MM cell	Enhances transferring from MSC to MM cells	[41]
B-cell lymphoma	Mitochondria, soluble factors	MSCs	Anti-apoptosis: upregulate BCL-2 family proteins Pro-oncogenic: Activation of NOTCH1 signaling	[46]
MDS	Proangiogenic factors (VEGF-A, IGFs, and EGFs) and mediators of fibrosis (LOXL, TGF-β, and LIF)	Bi-directional	MSCs adopt MDS-desirable features, Mitochondria dysfunction, genotoxic stress in HSCs, ↑ risk of developing to AML, Impaired myeloid and lymphoid differentiation in mice with MDS, Modulated expression of several cytokines in MSCs	[49]

### Gap junctions

Gap junctions (GJs) are comprised of two hemichannels (HCs) or connexons which in turn composed of arrays of connexin proteins. Ions, small metabolites, and organelles can be transferred through GJs. GJ intercellular communication (GJIC) in the HSC niche may lead to improvement of cellular bioenergetics, and rejuvenates the damaged recipient cells [50].

In normal hematopoiesis, mitochondria can transfer from BMSC to HSC using GJ by which GJs modulate granulopoiesis and differentiation to myeloid blood cell precursor. Hematopoietic stem/progenitor cells (HSPCs) quiescence and stemness are also determinable by GJ communication named as connexin-32 (Cx32). HSC quiescence retention and survival in the BM is dependent on CXCL12 secretion which is regulable by the expression of both connexin-43 (Cx43( and connexin-45 (Cx45) in MSC. During stress, Cx43 transfers ROS to BMSC which reduces HSC senescence [50].

Turning to malignancy, in co-culture with MM cells, MSCs exhibit an abrupt increase in Cx43 level representative of improved GJ-mediated intercellular communication [51]. Cx43 has also been postulated to be a putative player of adhesion and migration of MM cells demonstrated in primary MM cells and cell lines RPMI 8226, U266, and XG-7 which can finally increase cell proliferation and chemoresistance [18]. Adhesion and migration of MM cells is blocked by the gap junction blocker 18α-glycyrrhetinic acid (18α-GA) which decrease stromal cell-derived factor-1α (SDF-1α) secretion [51]. Divagating from MM, HL-60 and PBL-985 cells would see a downward trend in differentiation potential when GJ communication has not been abrogated [50, 52].

Carbenoxolone-induced GJ disruption could interfere with MSC and different malignant hematologic cell line communication and alters drug resistance pattern [53]. It indicates the important role of GJ between these cells in the outcome of leukemia treatment.

Cx26, Cx32, Cx37, Cx43, and Cx45 are responsible for exponentially elevated chemoresistance and substantially reduced apoptosis in primary AML cells. Leukemia pathogenesis is interconnected with connexin-based modifications on target cells. For example, mitochondria can transfer in a Cx43-mediated manner which affect adversely not only the pathogenesis but also chemoresistance [50]. Therefore, MSCs induced chemoresistance can be modulated by disruption of gap junctions in AML [53].

A significant amounts of prostaglandin E_2_ (PGE_2_), which suppresses DNA damage-induced p53 accumulation, is released from the HCs of stromal cells leading to promoted survival and metastasis of cancer cells [54]. However, having the permeability to small molecules and macromolecules, Cx43 may provide a target for cytoplasmic drug delivery [55]. Table 2 represents details of the GJ-mediated crosstalk between MSCs and malignant hematologic cells that has reported in different studies.

**Table 2:** Crosstalk between MSCs and hematologic malignancies/cell lines via gap junction and its effects

Type of malignancy/cell	Kind of gap junction's component(s) involved	Target	Results	References
HL-60 and PBL-985 cells	Cx43	HL-60 and PBL-985 cells	Downward trend in differentiation potential	[56]
U937, KG-1, KG-1a, HL-60, OCI-AML3, MV4-11, MoLM-13 Jurkat, and THP1 cells	Cx25, Cx26, Cx30, Cx31, Cx32, Cx36, Cx37, Cx40, Cx46, and Cx62	MSCs	Proliferation	[56]
Primary AML cells	Cx26, Cx32, Cx37, Cx43, and Cx45	AML cells	Chemoresistance, ↓ apoptosis	[50]
MM cells: RPMI 8226, U266, and XG-7	Cx43	MM cells	↑Cell proliferation, chemoresistance	[57]
AML	Cx43	MSCs	Pathogenesis, chemoresistance	[53]

## Contact-independent

### Paracrine activity

MSC produce cytokines such as IL-6, IL-11, SCF, TPO, Flt-3 ligand, CXCL12, G-CSF, GM-CSF and M-CSF [58–61] to support hematopoiesis. On the other hand, HSC quiescence is affected by Wnt released from MSC. Wnt expression in HSC also downregulated kit ligand, angiopoietin-1, CXCL12 and vascular cell adhesion molecule 1 (VCAM-1) [62, 63].

To turn to malignant hematopoiesis, BM-MSCs mainly produce Wnt ligands which leads to the intracellular accumulation of β-catenin. Gene expression of several downstream growth factors are subsequently elevated and proliferation of leukemia stem cells (LSCs) is guaranteed by this way [64, 65]. To elucidate, the effects of Wnt/β-catenin signaling as a pro-growth signal is essentially counteracted by the bone morphogenetic protein (BMP) anti-growth signals. Imbalance toward higher growth rate results in a leukemogenic phenotype [66]. However, dealing with Wnt/β‐catenin signaling, scientists have found this pathway to be complex and regulated by MSCs themselves. Interferon‐β (IFN‐β) released from MSCs exhibits anti‐tumorigenic effects in erythroleukemic cells based on its ability to negatively regulate Wnt/β‐catenin signaling pathways [67]. Dickkopf‐1 (DKK‐1) is also a negative regulator of Wnt signaling pathway and has antiproliferative activity in MM [68]. Considering the stimulatory effect of MSCs in production of DKK-1, IL-6, and IL-10, a potential role has been ascribed to the crosstalk between myeloma and MSCs in the development of disease into a bone lytic phase [57, 69]. Interestingly, in co-culture studies with multiple myeloma-derived mesenchymal stem cells (MM-MSCs), granulocytic-myeloid-derived suppressor cells (G-MDSCs) have been examined. MM-MSC educated G-MDSCs demonstrate supportive effects in MM by upregulation of immune-suppressive and proangiogenic factors including arginase 1 (ARG1), tumor necrosis factor α (TNF-α), and prokineticin 2 (PROK2) [70]. Besides, MSC ensures MM cell survival, disease progression, and drug resistance having upregulated levels of gene expression of angiogenic and growth factors such as CD40/40L, VCAM-1, ICAM-1, lymphocyte function-associated antigen-3 (LFA-3), and immunomodulated level of cytokines: increased IL-6 and reduced IL-10 [71]. Pro-angiogenic profile accompanied by anti-osteogenic pattern in MM cells co-cultivated by MM-MSCs can be a consequence of increased the vascular endothelial growth factor (VEGF) and IL-6 expression. As a matter of interest, this phenomenon is followed from activation of Notch signaling in MM-MSCs [72].

Regarding other cytokines produced by MSCs, they secrete promyelocytic leukemia protein followed by production of pro-inflammatory molecules, including CXCL1 and IL-6 which is considered as the major cause of leukemogenesis in the different types of leukemia [73]. For the most part, MSC secreted factors, especially IL-6, shelter CML cells from imatinib-induced apoptosis basically through NFκB-mediated signaling [74]. Chemoresistance in the diffuse large B-cell lymphoma (DLBCL) can be acquired by MSC secretion of IL-6 and upregulation of IL-17A [75].

In CLL, MSCs also demonstrate protective activities against cytotoxic effects of Forodesine [76]. To illustrate, interaction of MSC with CLL cells increases the production platelet-derived growth factor (PDGF), which binds to its receptor, PDGFR, leading to secretion of VEGF and making an angiogenic switch, associated with drug protection and disease progression [77]. Compared to MDS-derived mesenchymal stromal cells (MDS-MSCs), MSCs from B-CLL patients produce aberrant SDF-1, B-cell activating factor (BAFF), and transforming growth factor β (TGF-β) resulting in exponentially promoted normal B-cell proliferation and IgG production [78]. Elevated VEGF and hypoxia-inducible factor 1 (HIF-1) production is representative for proangiogenic profile and therefore additional CLL cell survival and resistance to rituximab/alemtuzumab [71].

Disease progression resulted from shifting to proangiogenic profile is the fatal outcome of interaction between conditioned medium (CM) obtained from CLL cells (CLL-CM) and MSCs. PDGFR in MSCs is converted to the active form after exposure to CLL-CM. Microenvironment must face devastating consequences of this phenomenon including MSC proliferation and MSC VEGF production [79]. Finally, survival of CLL cells is also insured by the interaction between their hepatocyte growth factor receptor (c-MET) and hepatocyte growth factor secreted by MSCs [80]. Some important MSCs-derived molecules and their related effects on HSCs and leukemic cells were shown in Table 3.

**Table 3:** Paracrine effects of MSCs on HSCs and different leukemic cells

Type of molecule released by MSC	Target	Results	References
Wnt	HSCs	Quiescence	[81]
IL-6, IL-11, SCF, TPO, Flt-3 ligand, CXCL12, G-CSF, GM-CSF, and M-CSF	HSCs	Ensures hematopoiesis	[82]
Wnt	HSCs	Downregulates kit ligand, angiopoietin-1, CXCL12, and VCAM-1	[82]
Wnt ligands	LSCs	Proliferation	[64, 65]
Wnt ligands	LSCs	Counteracted by BMP anti-growth signals	[66]
Interferon‐β (IFN‐β)	Erythroleukemic cells	Anti‐tumorigenic, negative regulation of Wnt/β‐catenin	[67]
Dickkopf‐1 (DKK‐1)	MM cells	Negative regulation of Wnt, development of disease into a bone lytic phase	[57, 68, 69]
↑CD40/40L, VCAM-1, ICAM-1, LFA-3, HO-1, IL-6, VEGF, and ↓ IL-10	MM cells/ endothelial cells	MM cell survival, disease progression, drug resistance, pro-angiogenic profile	[71, 72]
Promyelocytic leukemia protein (PML) protein	Different types of leukemic cells	CXCL1 and IL-6 production, leukemogenesis	[73]
IL-6	CML cells	Shelters CML cells from imatinib induced apoptosis	[74]
IL-6	Diffuse large B cell lymphoma	Chemoresistance, ↑IL-17A level	[75]
PDGF	CLL cells	Making an angiogenic switch, protective activities against cytotoxic effects of Forodesine	[76, 77]
SDF-1, BAFF, TGF-β	CLL cells	B-cell proliferation, IgG production	[78]
VEGF, HIF-1, HGF	CLL cells	Proangiogenic profile, CLL cell survival, resistance to rituximab/alemtuzumab	[71]

### Chemokines and bio active molecules

There are also some chemokines that are regulated by MSCs. First of all, through CXCL12-CXCR4 interaction between MSC and CML cells (respectively), imatinib-induced cell death is reduced as a consequence of attenuated caspase-3 activity [76]. Niches with CXCL12 devoid of MSCs, cannot support the LSCs from tyrosine kinase inhibitor (TKI) treatment, while CXCL12 + MSC niches offer a full guarantee for LSCs to maintain quiescent and TKI-resistant [83].

Secondly, CXCL8 derived from MSCs supports the survival and proliferation of AML cells through the PI3K/AKT pathway [84]. By the same token, via activation of NF-κB, MSC is involved in the residual disease maintenance in AML and on the other hand in therapy-resistance. This activation of NF-κB may be the consequence of interaction of VCAM-1 on MSC and its ligand, VLA-4, on leukemic cells [85]. Indeed, the underlying molecular mechanisms in BM niche by which the drug resistance and disease relapse are caused in AML include SDF-1/CXCL12, Wnt/β-catenin, VCAM/VLA-4/NF-κB, CD44, and hypoxia [86]. Axl is a member of the Tyro3 and has been approved of prognostic value and therapeutic target in AML that has been claimed as a mediator in the paracrine signaling between the leukemia cells and BM-MSCs. The expression of Axl ligand, growth arrest–specific gene 6 (Gas6), on MSCs can be elicited by AML cells.[87].

Other factors including Periostin (POSTN) is a multifunctional extracellular component. BM-MSC-derived POSTN promotes B-ALL cell-derived CCL2 which increases the leukemia burden [88]. Lumican (LUM) is an extracellular matrix protein secreted by MSCs. LSCs such as Nalm-6 (an ALL cell line) acquires anti-apoptotic properties and resistance to chemotherapy by downregulation of LUM expression in BM-MSCs [89].

Bone destruction in MM is mainly orchestrated by osteoclasts that undergo differentiation induced by the production of CCL3 and CCL4, matrix metalloproteinases (MMP)-13, IL-1, IL-3, IL-6 and IL-17 released by MSCs [72]. Conversely, AML cells shift the niche towards an osteoblastic one by the induction of connective tissue growth factor (CTGF) expression in BM-MSCs [90].

Co-cultured with MSCs, CML cells reduce caspase-3 activation and modulate Bcl-XL (anti-apoptotic protein) expression after treatment with imatinib which signify MSC-mediated protection of CML cells [91, 92]. This has been proven to be interceded with CXCR4/CXCL12; hence, combinational therapy with anti-CXCR4 antagonists and TKIs may represent a powerful approach in the treatment of CML [93].

Apart from intracellular signaling pathways, inhibiting the intercellular trafficking routes provides a promising therapeutic approach in leukemia. Using AMD3100 for example, the SDF-1α/CXCR4 axis is interrupted leading to hindrance of intercellular trafficking of CLL cells, and disturbance of microenvironment-mediated support [94]. AMD3100 is the first generation CXCR4 antagonist; therefore, it can inhibit proliferation of HSC and trafficking of leukocytes. However, BL8040 is the CXCR4 new generation inhibitor exhibiting higher affinity than AMD3100. As it has been presented in Table 4, chemokines and biomolecules interfere with malignant hematologic cells using different mechanisms.

**Table 4:** MSCs-secreted chemokines/biomolecules and their impacts on the hematologic malignancies

Kind of chemokine/biomolecule	Target	Results	References
CXCL12	AML cells	Dampening effect on MSC-mediated resistance to FLT3 inhibition	[95]
CXCL12	CML cells	↓Imatinib-induced cell death	[76]
CXCL12	LSCs of CML	Maintain quiescent of LSCs and TKI-resistant	[83]
Periostin	B-ALL cells	↑B-ALL cell-derived CCL2, ↑ leukemia burden	[88]
Lumican	LSCs of Nalm-6 cell line	Downregulation of anti-apoptotic, resistance to chemotherapy	[89]
CCL3, CCL4, matrix metalloproteinases (MMP)-13, IL-1, IL-3, IL-6, and IL-17	MM cells	Differentiation to osteoclast, bone destruction in MM	[72]
VCAM-1, SDF1, Wnt	AML cells	Residual disease maintenance, drug resistance and disease relapse	[85, 86]
Axl	AML cells	Prognostic factor and therapeutic target	[87]
SDF-1α/CXCR4	CLL cells	Intercellular trafficking of CLL cells	[94]
CXCR4/CXCL12	CML cells	↓Caspase-3 activation, Bcl-XL expression modulation after treatment with imatinib	[93]

### Microvesicles and exosomes

Microvesicles (MVs) and exosomes shed from MSCs membrane [10] and affect on different cell processes. Cell viability, clonogenic capacity and miRNA and gene expression profile of CD34^+^ cells in patients with MDS were all modified after receiving MVs derived from MSCs [49].

On the other hand, BM-MSCs ability to support CD34^+^ cells declines, after getting affected by extracellular vesicles (EVs) containing miR-7977 derived from AML/MDS CD34^+^ cells. miR-150 EVs target the CXCR4/SDF-1 axis which is fundamental for retention and differentiation of HSPC in BM. Instructed by human primary MDS cells, normal donor MSCs (ND-MSCs) adopt MDS-desirable features such as high expression of proangiogenic factors (VEGFA, IGFs, and EGFs) and mediators of fibrosis (LOXL, TGF-β, and LIF) [49].

Exosomes can be defined as the small, extracellular vesicles carrying a variety of biologic molecules, including proteins, DNA, mRNA and non-coding RNA. These proteins include antigen presenting molecules, adhesion molecules, membrane transport and fusion molecules, cytoskeletal proteins, pyruvate kinase, histones and others [10].

For the first consideration, MSC-derived EVs in the kidney, neurological, cardiovascular and liver diseases are of precious value that influence disease trajectory, patient survival and treatment strategy [96]. Secondly, cell-fate determination in stem cells is an EV-mastered process [97]. Modified by EVs, cancer stem cells (CSCs) and normal HSCs can develop and differentiate to various hematologic malignancies. These EVs are secreted by MSCs reprogrammed by CSCs and the neoplastic cells [98]. EVs content also modifies CD34^+^ cells viability as well as colony forming unit-granulocyte monocyte (CFU-GM) production. Precisely, some microRNAs like miR-10a and miR-15a are overexpressed in EVs from MSCs of MDSs patients and transferred to CD34^+^ cells. Modifying the expression of *MDM2* and *P53* genes, these microRNAs augment cell viability and increase clonogenic capacity [99]. On Immune cells, they induce immunosuppression [100]. EVs from MSC promote both proliferation and apoptosis of regulatory T cells [101]. They decrease Th17 cells and increase regulatory T cells on the peripheral blood mononuclear cells [102].

Based on studies on K562 cells-derived exosomes, these EVs may directly stimulate the target cells or transfer receptors between cells. They may deliver functional proteins and transfer the genetic materials like mRNA, miRNA, or transcription factors to target cell [103]. Another CML cell line, LAMA84, generates EVs that have effects on the human vascular endothelial cells leading to ICAM-1, VCAM‐1, and IL‐8 expression upregulation which indeed shift the tumor microenvironment (TME) to a pro-angiogenic pattern and therefore unfavorable prognosis [104].

Enhancement of angiogenesis is mainly mediated by the well-known pro-angiogenic factors such as VEGF, basic fibroblast growth factor (bFGF), and angiopoietin-1 secreted by the MM cells or stromal cells interacting with MM cells. Osteoclast differentiation and osteoclast bone resorption activity in MM is modulated and supported by MM cell-derived exosomes containing osteoclast activating factors which in turn enhance MM cell growth and survival by secretion of IL-6 and B-cell-activating factor [105].

Generally speaking, escaping from spontaneous or drug-induced apoptosis, migrating in higher rate and modifying genes more suitably are the main results of transferring EVs from leukemia patient MSCs compared to EVs from healthy donor MSCs [106]. IL-6 and IL-8 inhibit hematopoiesis by downregulating the CXCL12, angiopoietin 1, and kit ligand. In hematologic malignancies, IL-6 and IL-8 are upregulated in MSCs and are delivered to microenvironment by EVs [71].

LAMA84-derived exosomes promote IL-8 secretion in the MSC cell line, HS5, leading to enhance the survival, proliferation, and migration of LAMA84 cells in vitro. [71]. Based on a study about K562 cell, K562 exosomal miR-711 has been credited for suppressed adhesion abilities of BM-MSCs because of the fact that miR-711 is capable of silencing CD44—an adhesion molecule‒expression in BM-MSCs [107].

Exosomes from BM-MSCs contain miR-222-3p which is responsible for interferon regulatory factor 2/inositol polyphosphate 4-phosphatase type II (IRF2/INPP4B) signaling inhibition and has been greatly observed in co-culture with AML cell line [108]. IRF2/INPP4B signaling is involved in autophagy and apoptosis [109]. Exosomes secreted by AML cells alter the behavior of MSCs [110]. AML cell resistance to TKIs is effectively guaranteed by TGF-β1, miR-155, and miR-375 rich exosomes released by BM-MSC. Similarly, exosomes rich in miR-150 can disrupt the CXCR4/CXCL12 axis; disruption of CXCR4/CXCL12 axis supports the leukemia growth. These EVs are derived from AML cells and destined to be taken up by BM-MSCs [111].

Another in vitro study clarify that exosomes from AML cell lines HEL 92.1.7, HL-60, MOLM-14, and U937 transfer mRNA of insulin-like growth factor 1 receptor (IGF1R), matrix metalloproteinase 9 (MMP-9), nuclear matrix protein 1 (NPM1), CXCR4, and internal tandem duplication mutations in *FLT3* (FLT3-ITD) into BM-MSC [71].

By the same token, exosomes derived from MM-MSC aim to ensure disease progression in vivo by delivery of IL-6, CCL2, and fibronectin and by attenuating the expression of the tumor suppressor miR-15a [71].

Tax viral oncoprotein of human T-cell lymphotropic virus type I (HTLV-I) causes adult T-cell leukemia/lymphoma (ATL). BM-MSCs pick exosomes up from ATL cells containing the Tax oncoprotein and leading to reduced MSC stemness and improved angiogenesis due to the multiplied levels of VEGF, CXCR4, and MMP-9 [71]. IL-8 secretion is initiated by CML cell-derived exosomes and finally contributes to CML cell survival [112].

Mechanistically, it has been proposed that the content of tumor-suppressor miR-15a in MSC-EVs is determinative of the MSC communication with MM cells. Decreased miR-15a content in MM-MSCs induces tumor growth and promotes myeloma dissemination [113, 114]. Proliferation, cancer-associated fibroblast (CAF) transformation, and IL-6 secretion of MSCs increases in co-culture with MM cells and these have been partially guided by miR-21 and miR-146a delivered by MM cells [115]. Minimal residual disease (MRD) after treatment can be monitored by measuring the circulating EVs in MM. Remarkably, transforming from monoclonal gammopathy of undetermined significance into symptomatic myeloma can gain the advantage of predictability by identifying and measuring the circulating EVs [116].

Based on both in vitro and in vivo studies, the leukemia-surviving subpopulation of MSCs in CLL cells is created and developed following secretion of protein- and miRNA-containing exosomes by CLL cells [32]. MSCs from CLL patients support in vitro neoplastic B cell survival [117]

EVs would be brought highly on agenda considering the fact that tumor stage, risk of recurrence, drug resistance, and overall clinical outcome of patients correlate to a great extent with number, phenotype and the molecular content of EVs [116]. Table 5 indicates some of studies and their reports about MV-mediated communication of MSCs and malignant cells.

**Table 5:** Interaction between MSCs and hematologic neoplasms by microvesicles transferring

Type of malignancy/cell (source)	Target	Content	Results	References
MDS-MSCs	CD34^+^ cells	miR-10a and miR-15a	Modifying CD34^+^ cell viability, CFU-GM production, *MDM2* and *P53* genes expression	[99]
CML cell line LAMA84	Human vascular endothelial cells	Different biomolecules	Upregulation of ICAM‐1, VCAM‐1 and IL‐8, pro-angiogenic pattern	[104]
MM	MSCs	Osteoclast activating factors	Osteoclast differentiation, osteoclast bone resorption activity	[105]
MM	MSCs	miR-21 and miR-146a	MM cell growth, survival and proliferation, CAF transformation, IL-6 secretion of MSCs	[115]
B-CLL	Leukemia B cells	CCL3/4, EGR1/2/3, and MYC	Escaping from spontaneous or drug-induced apoptosis, migrating in higher rate and modifying genes more suitably	[106]
MSCs	Microenvironment	IL-6 and IL-8	Hematopoiesis inhibition by downregulating the CXCL12, angiopoietin 1, and kit ligand	[71]
MSC cell line HS5	CML cell line LAMA84	IL-8	Survival, proliferation, migration	[71]
K562	BM-MSCs	miR-711	Suppressed adhesion abilities of BM-MSCs	[107]
MSCs	AML cell line	miR-222-3p	IRF2/INPP4B signaling inhibition	[108]
MSCs	AML cell line	TGF-β1, miR-155, and miR-375	AML cell resistance to tyrosine kinase inhibitors	[111]
AML	MSCs	miR-150	Disruption of the CXCR4/ CXCL12 axis	[111]
AML cell lines; HEL 92.1.7, HL-60, MOLM-14, and U937	MSCs	mRNA of IGF1R, MMP-9, NPM1, CXCR4, FLT3 FLT3-ITD	Leukemia progression	[71]
MM-MSC	MM	IL-6, CCL2 Fibronectin	↓ miR-15a content in MM-MSCs, induces tumor growth and promotes myeloma dissemination	[71, 113, 114]
ATL	MSCs	Tax viral oncoprotein of HTLV-I	↓ MSC stemness and improved angiogenesis	[71]
CML	MSCs	IL-8	IL-8 secretion, CML cell survival	[112]
CLL	MSCs	Protein and miRNA	Creation of leukemia-surviving subpopulation of MSCs	[117]

## Discussion

MSCs could communicate with malignant hematologic cells by different contact-dependent and/or contact-independent mechanisms. Notably in CML, TNTs facilitate exosomes transportation and exosomes in turn stimulate TNT formation; so, they can synergistically interact and multiply their communication [45]. However, having permeability to small molecules and macromolecules, Cx43 may provide a target for the cytoplasmic drug delivery [55]. Inhibition of oxidative phosphorylation (Oxphos) pathway in mitochondria also contributes to drug-resistance of AML based on the fact that TNT formation and mitochondrial transfer from BM-MSCs to AML is facilitated and promoted in this way [118]. In Jurkat cells, MSC-induced chemoresistance can be controlled by inhibition of mitochondrial transfer [36]. On the other hand, TNT formation is downregulated by NF-κB inhibitor BAY-117082 in AML [119]. Currently, BM-MSCs are found to be able to significantly enhance the drug resistance to various chemotherapy drugs, such as vincristine and cytarabine in ALL cells [120].

To clarify, in TME, MSCs-derived MVs can block the anti-tumor activity on immune cells and/or converts them into suppressor cells [111]. Another ascribed anti-tumor activity to MSCs is restoration of BM microenvironment via reprogramming the host macrophages [121]. Furthermore, MSC can inhibit the responses to alloreactive T lymphocytes as well as proliferation and cytotoxicity of natural killer (NK) cells [122].

Surprisingly, MSCs play an essential role for leukemia progression and chemoresistance by mitochondrial transfer, though the fate of transferred mitochondria in leukemic cells remains unclear. MSCs from patients with MDS and AML have a wide range of chromosomal aberrations, genetic and transcriptomic alterations. Deficiency of focal adhesion kinase (FAK) in MDS-MSCs correlates with ineffective hematopoiesis as it regulates the adhesion and mobility of cells [123].

It has been by the way proposed that the quiescence of AML blasts is ensured and outlasted in coculture with MSC resulting in increased leukemic survival in the presence of cytarabine [124]. Primary human AML cells remain proliferative for long-term by growth-enhancing effects of normal MSCs which is mediated by increased phosphorylation of the mammalian or mechanistic target of rapamycin (mTOR) and its downstream targets [125]. Diminished apoptosis is representative of tumor promoting effects of MSCs on MM cells and generally results from downregulation in caspase‐3 and poly (ADP‐ribose) polymerase expression which is associated with and mediated by enhanced AKT and ERK activities in MM cells [126].

## Conclusions

In summary, bidirectional relationship between MSCs and hematologic malignancy-derived cells has different contact-dependent and contact-independent mechanisms. These cross-talks affect disease progression and outcome. The fate of malignant cells, drug resistance conditions, MRD status and other cellular processes are regulated by the MSC behavior. There are many studies conducted to understand the exact underlying mechanisms of MSCs and malignant hematologic cells communication. Their results could be applicable to design an improved treatment protocol and ameliorated patient’s survival. Hence, focus on this field and conducting additional studies or review with more confirmed information are emphatically suggested in this regard. We finally can infer that MSC does not behave similarly against different malignant hematologic cells and it basically extracted from the diverse responses and signals emitted from MSC in TME. It seems that the nursing role of MSCs in one hematologic neoplasm may be reversed in another by tumor progression and anti-apoptotic benefit.

## Data Availability

Not applicable.

## Abbreviations

MSCs: Mesenchymal stem/stromal cells
BM-MSCs: Bone marrow-derived mesenchymal stromal cells
TNTs: Tunneling nanotubes
miR: MicroRNA
MI: Myocardial infarction
CLL: Chronic lymphocytic leukemia
BMSCs: Bone marrow stromal cells
SDS: Schwachman–Diamond syndrome
DDR: DNA damage response
B-ALL: B cell acute lymphoblastic leukemia
BCP-ALL: B-cell precursor ALL
ICAM1: Intercellular adhesion molecule 1
IP-10: Interferon-γ-inducible protein 10
MCP-1: Monocyte chemotactic protein-1
T-ALL: T cell acute lymphoblastic leukemia
GJs: Gap junctions
ARA-C: Cytosine arabinoside
ROS: Reactive oxygen species
AML: Acute myelogenous leukemia
NOX2: NADPH oxidase 2
MM: Multiple myeloma
CML: Chronic myeloid leukemia
HCs: Hemichannels
GJIC: GJ intercellular communication
HSPCs: Hematopoietic stem/progenitor cells
Cx: Connexin
SDF-1α: Stromal cell-derived factor-1α
PGE_2_: Prostaglandin E_2_
VCAM-1: Vascular cell adhesion molecule 1
BMP: Bone morphogenetic protein
LSCs: Leukemia stem cells
DKK‐1: Dickkopf‐1
MM-MSCs: Multiple myeloma-derived mesenchymal stem cells
G-MDSCs: Granulocytic-myeloid-derived suppressor cells
ARG1: Arginase 1
TNF-α: Tumor necrosis factor α
PROK2: Prokineticin 2
LFA-3: Lymphocyte function-associated antigen-3
VEGF: Vascular endothelial growth factor
DLBCL: Diffuse large B-cell lymphoma
PDGF: Platelet-derived growth factor
MDS-MSCs: MDS-derived mesenchymal stromal cells
BAFF: B-cell activating factor
TGF-β: Transforming growth factor β
HIF-1: Hypoxia-inducible factor 1
CM: Conditioned medium
TKI: Tyrosine kinase inhibitor
Gas6: Growth arrest–specific gene 6
POSTN: Periostin
LUM: Lumican
MMP: Matrix metalloproteinase
EVs: Extracellular vesicles
ND-MSCs: Normal donor MSCs
CSCs: Cancer stem cells
CFU-GM: Colony forming unit-granulocyte monocyte
TME: Tumor microenvironment
bFGF: Basic fibroblast growth factor
IRF2/INPP4B: Interferon regulatory factor 2/inositol polyphosphate 4-phosphatase type II
IGF1R: Insulin-like growth factor 1 receptor
FLT3-ITD: Internal tandem duplication mutations in FLT3
HTLV-I: Human T-cell lymphotropic virus type I
ATL: Adult T-cell leukemia/lymphoma
CAF: Cancer-associated fibroblast
MRD: Minimal residual disease
FAK: Focal adhesion kinase
Oxphos: Oxidative phosphorylation
mTOR: Mammalian (mechanistic) target of rapamycin
